# Junín virus induces autophagy in human A549 cells

**DOI:** 10.1371/journal.pone.0218730

**Published:** 2019-06-19

**Authors:** Maria Laura A. Perez Vidakovics, Agustín E. Ure, Paula N. Arrías, Víctor Romanowski, Ricardo M. Gómez

**Affiliations:** Instituto de Biotecnología y Biología Molecular, CONICET-Universidad Nacional de La Plata, La Plata, Argentina; Univerzitet u Beogradu, SERBIA

## Abstract

Autophagy, a highly regulated degradative process that promotes cellular homeostasis, is increasingly recognised as a fundamental component of the cellular response against viral infection. In this study, we investigated the role of autophagy during Junín virus (JUNV) multiplication using human A549 cells. We found that JUNV infection induces an increment of the LC3-II/LC3-I ratio, an accumulation of punctate pattern in RFP-LC3-transfected cells and the colocalisation of viral nucleoprotein and LC3 protein, suggesting autophagosome formation. JUNV infection also induced the degradation of the autophagy receptor p62, suggesting that complete autophagic flux was triggered. In addition, we showed that inhibition of autophagy with bafilomycin A1 or 3-methyladenine significantly reduces viral multiplication. Moreover, viral yield was increased when autophagy was induced using rapamycin. Furthermore, JUNV infection induced the colocalisation of p62, ATG16, RAB5, RAB7A and LAMP1 with the autophagosomal LC3 protein. That suggests that phagosomes undergo the maturation process during viral infection. Finally, we demonstrated that siRNA experiments targeting essential autophagy genes (*ATG5*, *ATG7* and *Beclin 1*) reduce viral protein synthesis and viral yield. Overall, our results indicate that JUNV activates host autophagy machinery enhancing its multiplication.

## Introduction

Autophagy is a highly conserved and tightly regulated process in which cellular components are engulfed into double-membrane vesicles (autophagosomes) and degraded to maintain cellular homeostasis. This process begins with a negative membrane curvature which expands and encloses the cytosolic cargo, forming the autophagosome, that later fuses with late endosomes or lysosomes for degradation [1,2]. The initiation of autophagosome formation is negatively regulated by the mammalian target of rapamycin complex 1 (mTORC1) kinase. When the mTORC1 complex is inhibited, the ULK1 kinase complex summons autophagy-related (ATG) proteins to the site of nucleation of the autophagosome precursor (phagophore) [3]. Downstream of ULK1, other important autophagy components exist, such as the phosphatidylinositol 3-kinase (PI3K) complex (which includes Beclin 1 and VPS34) and the ATG12 (ATG12–ATG5-ATG16) and LC3 complexes [4]. The activation of PI3K results in the recruitment of the ATG12 complex to isolation membranes and the lipidation of the LC3. This lipidated form of LC3, known as LC3-II, is associated with the internal and external surface of the expanding phagophores and is generally used as a marker of autophagosomes [5]. The association of LC3-II to the membrane is related to the expansion of the isolation membrane and closure of autophagosomes. After fusion with late endosomes or lysosomes, the autophagosome becomes an autolysosome, where LC3-II is partly degraded and partly delipidated and recycled [6].

Functional autophagy is essential for cell viability under stress conditions, including viral infections [7,8]. This cellular process can be antiviral or proviral, depending on the virus and the cellular context. A central aspect of the autophagic antiviral activity is to promote viral clearance by degradation of viral components. Moreover, autophagy also contributes to the innate and adaptive immunity by boosting the delivery of Toll-like receptor ligands to endosomes or by feeding antigens to the MHC class II pathway [7,9]. On the other hand, some viruses take advantage of autophagy by using elements of the autophagic machinery to ensure a proper cellular platform for replication [10–12] or by benefiting from the rearrangement of cellular lipid metabolism in order to support stronger viral replication [13]. Therefore, the interaction of viruses with the host autophagy machinery may be a crucial mechanism for cell survival and could determines infection outcome.

The family *Arenaviridae* currently includes two genera, *Mammarenavirus* (mammals) and *Reptarenavirus* (reptiles) [14]. On the basis of serological, geographical and genetic evidence, the mammarenaviruses are subdivided into Old World (OW, Africa, Europe, and Asia) and New World (NW, Americas) group [15]. The mammarenaviruses are generally associated with infection in rodents, but some are responsible for fatal diseases in humans, like Argentine hemorrhagic fever (AHF) caused by Junín virus (JUNV) [16]. Therefore, the hemorrhagic fever-causing mammarenaviruses are recognised to pose a significant threat to public health and are classified as category A priority pathogens [17]. Arenaviruses are enveloped and pleomorphic, with a diameter of 60–300 nm and two single-stranded RNA genome segments with an ambisense coding strategy [18]. The bipartite genome encodes four proteins: the matrix protein Z, the RNA-dependent RNA polymerase L, the major nucleocapsid protein N, and the glycoprotein precursor GPC [18]. Viral entry of NW clade B viruses is mediated by the transferrin receptor 1 (TfR1) [19], although it has been shown that JUNV also employs alternative cell-surface molecules [20]. Moreover, it has been demonstrated that clathrin-mediated endocytosis is the main route used by JUNV and involves the cytoskeleton and other cellular proteins [21,22]. Genome release into the cytoplasm depends on the pH-dependent fusion of the viral and endosomal membranes, a process mediated by the virus envelope G2 protein, as part of the glycoprotein complex [23]. In particular, JUNV internalisation leads to PI3K/Akt signalling pathway activation [24] and requires both actin and a dynamic microtubule network [25]. However, the interplay between the mechanisms and regulation of intracellular trafficking and the arenavirus life cycle remains mostly unexplored.

In individuals with AHF, the severity and prognosis of the disease correlate with high levels of IFN [26]. Several proteins involved in the IFN signalling mechanisms are also implicated in the regulation of autophagy [27]. Moreover, recent studies show that autophagy plays a critical role in the production of IFN during viral infection [28,29]. Therefore, we aimed to investigate whether JUNV can modulate the host autophagic processes and how autophagy affects JUNV infection. Studying the subcellular distribution of autophagosome markers and using fluorescent-tagged probes to monitor the autophagy flux, we found that JUNV infection induces autophagy in A549 cells. Moreover, we used pharmacological inducer/inhibitors of autophagy and small interfering RNAs targeting critical components of the autophagic machinery and analysed the effect on JUNV multiplication. Our results suggest that JUNV exploits the host autophagy to enhance its multiplication.

## Materials and methods

### Antibodies and reagents

For the detection of Junín virus, rabbit polyclonal antibody against JUNV N protein [30] or mouse monoclonal antibodies (mAb) obtained through BEI Resources, NIAID, NIH: Monoclonal anti-Junin Virus, Clone SA02-BG12 (produced *in vitro*), NR-49274 and Clone NA05-AG12 (produced *in vitro*), NR-48834, were used. Rabbit anti-LC3 (L7543) polyclonal antibodies (pAb) and rapamycin (Rap, R0395) were purchased from Sigma-Aldrich. Bafilomycin A1 (BAF, 1334) and 3-methyladenine (3-MA, 3977) were purchased from Tocris Bioscience. Rabbit anti-β-actin pAb (ab8227) was purchased from Abcam. Rabbit anti-LC3B (3868), anti-RAB5 (3547), anti-LAMP1 (9091), anti-Beclin 1 (3495), anti-ATG5 (12994), anti-ATG7 (8558), anti-ATG16L1 (8089) and anti-RAB7A (9367) mAbs were purchased from Cell Signalling Technology. Mouse anti-p62/SQSTM1 mAb was acquired from R&D Systems (MAB8028). Alexa Fluor 488-conjugated donkey anti-mouse IgG (715-545-150), Cy3-conjugated donkey anti-rabbit IgG (711-165-152) and Alexa Fluor 488-conjugated donkey anti-rabbit IgG (711-545-152) were purchased from Jackson ImmunoResearch. Plasmid RFP-LC3 was kindly provided by Dr Maria Isabel Colombo. pBABE-puro mCherry-EGFP-LC3B was a gift from Jayanta Debnath (Addgene plasmid # 22418).

### Viruses

Viral stocks of the virulent P3441 strain [31] of JUNV and TCRV were amplified as previously described [31]. Briefly, monolayers of BHK21 cells were infected with each strain and clarified supernatants (5,000 × g) were collected 4 to 5 days post-infection (d p.i.). UV inactivation of viral stocks was performed by irradiation at 254 nm using a UV Lamp (CAMAG) for 1 h as described [32]. Samples of virus stocks UV-inactivated were analysed by plaque assay as described below, to ensure complete inactivation. Viral manipulations were conducted in a biosafety cabinet class II type A2 under biosafety level 3 (BSL3) conditions. All laboratory personnel is vaccinated with the *Candid*#*1* vaccine.

### Cell culture and virus infection

A549 (ATCC No. CRM-CCL-185), Vero E6 (ATCC No. CRL-1586) and BHK-21 (ATCC No. CCL-10) cells were grown in minimum essential medium (MEM, Life Technologies) containing 10% fetal bovine serum (FBS, Life Technologies). Cells were incubated at 37°C in a 5% CO_2_ incubator. All cell cultures were tested for the presence of Mycoplasmas using a PCR detection kit (8208) from ScienCell Research Laboratories. For virus infection experiments, A549 cells cultured in 24 or 6-well plates were infected with JUNV or TCRV at a MOI of 3 and the virus inoculum was removed after adsorption for 1 h. The cell monolayers were incubated in complete fresh medium at 37°C for the indicated times, afterwards supernatants and cells were harvested. Optimal concentrations of different drugs (3-methyladenine, bafilomycin A1 and rapamycin) were used to explore the effects of autophagy on the multiplication of JUNV. Briefly, A549 cells were pretreated for 3 h with the indicated drugs prior to JUNV or mock infection. Subsequently, the cells were infected with JUNV as above and further incubated in fresh media in the absence or presence of these drugs at the same concentrations as for the pretreatments. Corresponding DMSO or _dd_H_2_O were used as vehicle controls. At the indicated times, supernatant and cells were collected and analysed for viral titre and protein expression as described below.

### Transfection

A549 cells were seeded on glass coverslips in 24-well tissue culture plates (Corning Glass Works, Corning). The following day, the cells were transfected at 60% confluence with the plasmid at 3,0 μg/well using the transfection reagent (Roche) according to the manufacturer’s instructions. The medium was replaced after 6 h with MEM containing FBS, and the cells were cultured for another 24 h before infection.

### Western blot analysis

Western blot analysis was performed as previously described [33]. Briefly, the cells were washed with PBS for three times and then scraped off, incubated on ice with cell lysis buffer (150 mM NaCl, 50 mM Tris–HCl pH 7.4, 1 mM EDTA, 1% N-40) containing a protease inhibitor cocktail (Roche Molecular Biochemicals) and 0.1 mM PMSF for 2 h. The cell lysates were centrifuged at 14,000 × g for 20 min at 4°C. The protein concentration was determined using the Bradford assay. Equal amount of protein samples were diluted in 6 × Laemmli sample buffer and separated on SDS-PAGE gels. The proteins in the gel were transferred to polyvinylidene fluoride (PVDF) membranes (Millipore, ISEQ00010) which were then blocked with 5% non-fat dry milk in TBST for 2 h and incubated overnight at 4°C with the primary antibodies. The membrane was then incubated for 2 h with the appropriate secondary antibodies. Immunoreactive bands were visualised using the enhanced chemiluminescence system (ECL, PerkinElmer Life Sciences). The intensities of the Western blot bands were analysed using ImageJ software.

### Plaque formation assay

Ten-fold dilutions (from 10^−1^ to 10^−3^) of the viral culture supernatants were added to 24-well plates with a 40–50% confluence monolayer of Vero E6 cells. The plate was then incubated at 37°C for 1 h with gentle rocking. Following adsorption, the inoculum was removed and overlaid with 2 ml of MEM containing 0.8% methylcellulose and 2% FBS and further incubated at 37°C in a humid atmosphere with 5% CO_2_. Plaques were allowed to develop for either 4–6 days before being fixed (4% w/v paraformaldehyde) and stained with a 1% Crystal Violet in 20% ethanol and _d_H2O.

### Confocal microscopy

For confocal imaging, the cells were grown on glass coverslips and then infected with JUNV as indicated at a MOI of 3. At the indicated h p.i, the cells were washed once in PBS and fixed with 4% PFA for 15 min at room temperature (RT). To stain endogenous LC3, fixed cells were permeabilised in 100% cold methanol for 10 min at −20°C, washed three times in PBS and incubated in blocking buffer (2% FBS, 1% normal goat serum in PBS) for 1 h at RT as previously described [34]. Alternatively, fixed cells were permeabilised with 0.1% Saponin-5% normal goat serum in PBS for 15 min. Immunostaining of LC3 was performed overnight at 4°C by incubating cells in primary antibody solution, then washed with PBS and followed by 2 h incubation with the corresponding secondary antibody. To detect colocalisation of LC3 and RAB5, RAB7A or LAMP1 primary antibodies and their respective secondary antibodies were used as indicated. The nuclei were stained with 4′-6-Diamidino-2-Phenylindole (DAPI, D9542, Sigma Aldrich). Coverslips were mounted with Prolong gold and stored at 4°C in the dark until observation. The confocal images were collected on a Leica TCS SP5 II microscope. This microscope was equipped with an HCX PL APO CS 63.0x 1.40 oil UV objective, a 543 nm line of an helium/neon laser, a 488 nm line of an argon/ion laser and LAS AF version 2.2.1 4842 software. Images were processed using ImageJ software (NIH). For colocalisation analysis, Pearson's correlation was calculated using “Coloc 2” tool [35]. Pearson’s score above 0.5 was considered a strong co-localization [36].

### RNA interference

A549 cells were grown to 60% confluence in 6-well cell culture plates and transfected with siTran transfection reagent (TT300001, Origene) following the manufacturer's recommendations. A final concentration of 5 nM siRNA against *BECN1* (SR305711), *ATG5* (SR306286), *ATG7* (SR307159) or *RAB7A* (SR305302) was used (Origene). The efficient knockdown of the target protein was evaluated by WB.

### Statistical analysis

The results were expressed as the mean ± s.d. of three independent experiments. Student's unpaired *t*-test was used to evaluate the difference between the test samples and controls. A p-value <0.05 was considered statistically significant.

## Results

### LC3 accumulation is induced by JUNV infection

**Fig 1:**
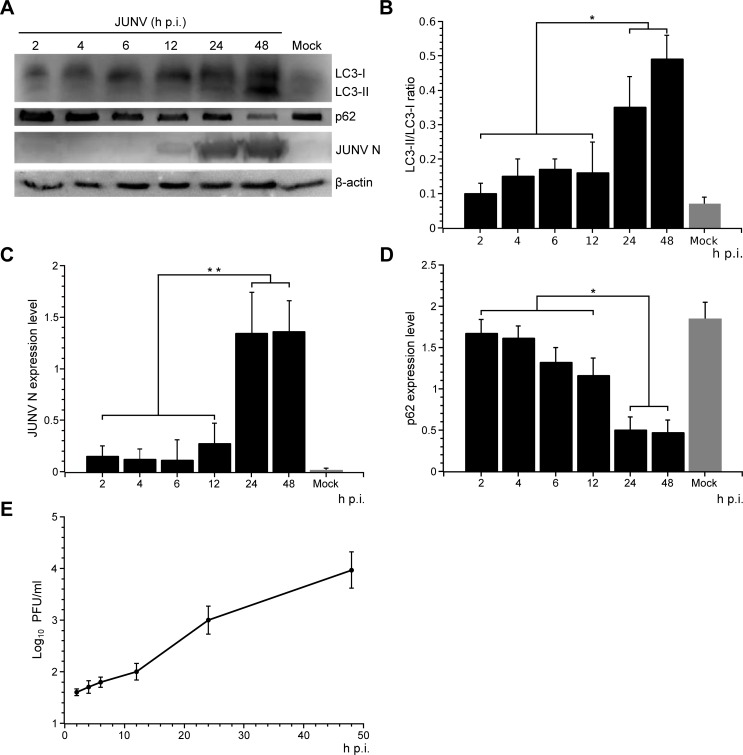
JUNV infection correlates with the accumulation of the LC3 autophagy marker protein. (A) JUNV infected A549 cells were harvested at indicated time points, and WB was performed to detect LC3, p62 and JUNV N. β-actin protein expression was assessed as control. (B-D) The WB bands were quantified by densitometric analysis using ImageJ software in order to calculate the JUNV N, LC3-II, LC3-I and p62 expression levels and were normalised to the β-actin loading control. Results represent the mean data from three independent experiments. *, P < 0.05. Full-length blots are presented in S1 Fig. (E) Determination of viral titre of the cell supernatants by plaque formation assays (PFU/ml) on Vero E6 cells. The data correspond to the mean ± s.d. (n = 3).

Several approaches need to be combined in order to reliably demonstrate the autophagy status of human cell lines. Established assays used to monitor autophagy include biochemical detection of the conversion of the cytosolic LC3-I to membrane-bound lipidated LC3-II by immunoblotting, observation of changes in subcellular distribution of autophagosomal protein markers by immunofluorescence (e.g. LC3 puncta formation), and detection of autophagosomes using fluorescent-tagged probes to monitor autophagy flux [5,37]. In order to assess autophagosome formation, we examined LC3 status after infection of A549 cells with JUNV. Protein samples from harvested cells at increasing times p.i. were subjected to Western blot (WB) analysis using an anti-LC3 antibody that recognises both forms of LC3 (Fig 1A and 1B). Simultaneously, rabbit antibodies against N were used to track the progression of infection (Fig 1A and 1C). The expression level of LC3-II was significantly increased from 12 to 24 h p.i. in JUNV infected cells compared to the mock, indicating the activation of autophagy during JUNV infection of A549 cells. In parallel, JUNV N increased sharply 24 h p.i., which was consistent with the increase of LC3-II and the progression of infection (Fig 1A, 1C and 1E). In contrast, no significant change in the LC3-II level was observed in mock-treated cells at 48 h. To establish if JUNV infection altered the autophagic flux involving the turnover of autophagosomal proteins, we analysed the expression level of p62, a polyubiquitin-binding protein (also known as Sequestosome-1 (SQSTM1)), which interacts with LC3 [38]. The p62 and p62-bound polyubiquitinated proteins become incorporated into the completed autophagosome and are degraded in autolysosomes [37], thus serving as an indicator of autophagic degradation. Inhibition of autophagy correlates with increased levels of p62 in mammals, suggesting that steady-state levels of this protein reflect the autophagic status [37]. Immunoblotting analysis revealed significant progressive degradation of p62 in JUNV infected cells compared to mock-infected cells (Fig 1A and 1D), suggesting that autophagosomes were able to fuse with lysosomes to degrade the cargos. Taken together, these data suggest that the autophagic flux in JUNV infected cells is activated and remains complete.

### JUNV nucleoprotein colocalises with LC3 positive vesicles

**Fig 2:**
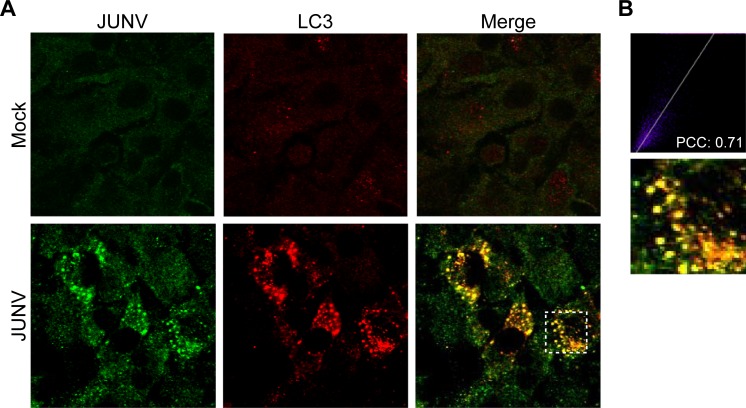
Viral N protein colocalises with the autophagy protein LC3 in JUNV-infected A549 cells. (A) Representative confocal microscopy images show mock or JUNV infected A549 cells. Cells were fixed and processed for IF 24 h p.i. LC3 was detected using a mAb rabbit anti-LC3B from Cell Signaling Technology and a pAb Cy3-conjugated donkey anti-rabbit IgG as secondary Ab. JUNV N was detected using a mAb mouse anti-JUNV N from BEI Resources followed by incubation with Alexa Fluor 488-conjugated donkey anti-mouse IgG as secondary antibody. (B) Images were analysed using Image J software. A higher-magnification view was included. Pearson’s coefficient was used for assessing colocalisation.

During autophagy, LC3 can be redistributed from a diffuse cytoplasmic localization to a distinctive punctate cytoplasmic pattern, which reveals the recruitment of LC3 to autophagic vesicles [5,39]. In order to assess the localization of endogenous LC3, A549 cells were infected with JUNV and after 24 h p.i. the cells were fixed and subjected to immunoconfocal microscopy analyses. The localization of viral proteins in infected cells was analysed using a specific monoclonal antibody against JUNV N. LC3 punctate pattern colocalised with N in JUNV infected cells, pointing out that viral nucleoprotein was associated with autophagic membranes during infection (Fig 2).

### Pharmacological inhibition of autophagy decreases JUNV multiplication

**Fig 3:**
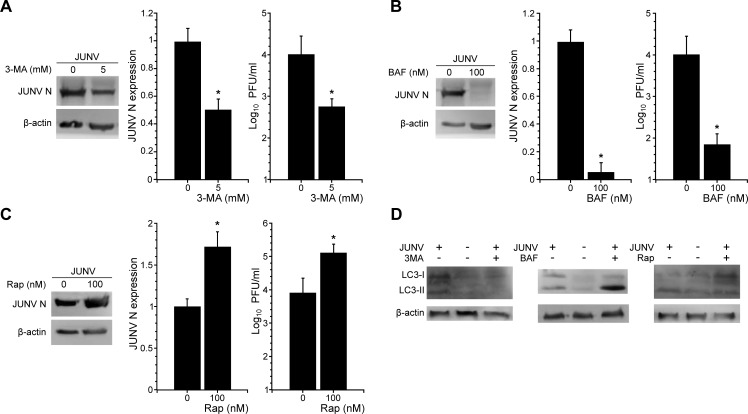
Effects of pharmacological modulation of autophagy on JUNV infection. A549 cells were pretreated or not with (A) 3-MA (5 mM), (B) bafilomycin A1 (BAF, 100 nM) or (C) rapamycin (Rap, 100 nM) for 3 h, infected with JUNV at MOI = 3 and cultured in MEM containing the corresponding drugs at the same concentration as for the pretreatment. WB analysis of JUNV N and β-actin (control) expression was performed at 24 h p.i. JUNV N/β-actin expression ratio was established by densitometry using ImageJ software. Viral titre from cell supernatants was determined using plaque-forming assay on Vero E6 cells. The data correspond to the mean ± s.d. (n = 3); Student's t-test; *, P <0.05. (D) In parallel experiments, cells pretreated with 3-MA (5 mM), bafilomycin A1 (BAF, 100 nM) or rapamycin (Rap, 100 nM) followed by mock (control) or JUNV infection were analysed by WB to detect LC3. β-actin protein expression assessed as control. Full-length blots are presented in S2 Fig.

Given that autophagy is increased during JUNV infection, we then determined whether cellular autophagy regulates JUNV multiplication. To accomplish this, we pretreated A549 cells with 3-methyladenine (3-MA), an autophagy inhibitor acting on the PI3K pathway [40]. As indicated by WB analysis, treatment of cells with 3-MA (Fig 3A) reduced the expression of N in infected cells when compared to untreated cells. Moreover, a decrease in viral progeny in the supernatant medium of the cells treated with 3-MA was confirmed by titration of infectious particles (Fig 3A). To ensure that 3-MA was inhibiting autophagy, LC3-II was assayed in parallel samples (Fig 3D).

Next, bafilomycin A1 (BAF) was used to analyse the turnover of autophagosomes induced by JUNV infection. BAF is an inhibitor of the vacuolar (V)-type ATPase, which avoids the fusion between autophagosomes and lysosomes and thereby prevents maturation of autophagosomes [41]. JUNV or mock infected A549 cells were pre-treated with BAF for 3 h. The expression of JUNV N was determined at 24 h p.i. by WB (Fig 3B). A decrease of N expression in infected cells was observed in response to BAF treatment. Furthermore, we observed a significant decrease of the viral titre in the supernatant of JUNV infected cells treated with BAF (Fig 3B). As expected, increased LC3II levels were detected, confirming the inhibition of autophagic degradation by BAF (Fig 3D).

**Fig 4:**
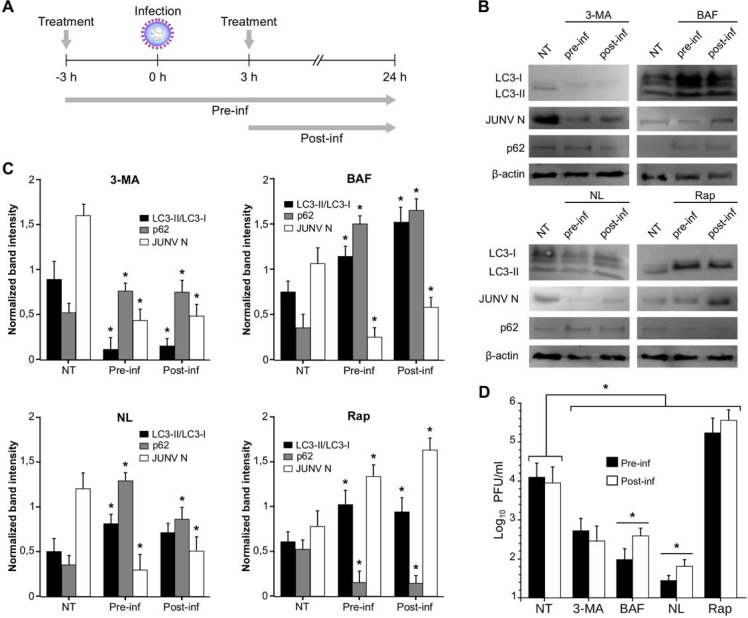
Pharmacological modulation of autophagy affects early or late stage of JUNV multiplication. (A) Experimental design showing the times of infection, treatment (autophagy inhibitors or inducers addition), and times of sample collection. At 3 h pre-infection or 3 h post infection with JUNV, A549 cells were treated for with 3-MA (5 mM), bafilomycin A1 (BAF, 100 nM), NL (ammonium chloride 20 mM, 100 μM Leupeptin) or rapamycin (Rap, 100 nM). After infection, Cells were further incubated in MEM containing the corresponding drug (for the indicated time). (B) Pre-infection or post-infection treated cells as indicated in (A) were collected, and WB analysis of JUNV N, LC3, p62 and β-actin (control) expression was performed at 24 h p.i. (NT: no treatment, JUNV infected cells). Full-length blots are presented in S3 Fig. (C). The expression levels of JUNV N, LC3-II, LC3-I and p62 were established by densitometry using ImageJ software and normalised to the β-actin loading control. The data correspond to the mean ± s.d. (n = 3); Student's t-test; *, P <0.05. (D) Determination of viral titre of the cell supernatant from experiments performed as indicated in (A) by plaque formation assays (PFU/ml) on Vero E6 cells. The data correspond to the mean ± s.d. (n = 3); Student's t-test; *, P <0.05.

The blockage of endosomal acidification negatively affects the infectivity of viruses that require a low pH for membrane fusion and release of the viral genome. In order to dissect the stage of viral cycle targeted by each one of the autophagy inhibitors, cells were either treated with the different drugs three hours before the infection (viral adsorption) or three hours after the infection (Fig 4A). Later, they were cultured in MEM containing each drug at the same concentration as for the treatment. After 24 h p.i., the viral titre from cell supernatant and the expression levels for p62, N and the LC3-II/LC3-I ratios were assessed. Either with the early autophagy inhibitor (3-MA) or the late inhibitor (BAF), the treatment pre or post-infection resulted in reduced expression of N and lower viral titre as compared to JUNV-infected cells that received no treatment (NT) (Fig 4B–4D). Additionally, a combination of ammonium chloride and leupeptin (NL) treatment to inhibit total lysosomal proteolysis also resulted in a decrease of N expression and viral production in infected cells. Together, these results show that inhibition of lysosomal degradation reduces infectious viral progeny. Importantly, the effect of BAF and NL treatments was clearly observed upon addition of drugs 3 h post-infection, when attachment, internalisation and uncoating have been completed (viral yield reduction: BAF, 51% (pre-infection) and 34% (post-infection); NL, 64% (pre-infection) and 54% (post-infection)). This result would indicate that inhibition by these compounds is at least partially targeted to events that take place after virus uncoating.

### Viral yield is enhanced by the induction of autophagy

To further determine the involvement of autophagy during JUNV infection, we investigated the effect of autophagosome induction using rapamycin (Rap), which induces autophagy by blocking the mTOR pathway [42]. Under these conditions, viral yields of JUNV infected cells were higher than in untreated cells (Fig 3C). In parallel, experiments performed adding Rap post-infection resulted in a similar increase in viral yields of JUNV, which indicates that the activation of autophagy benefits both viral entry/uncoating and later steps as assembling and viral budding (Fig 4). The effect of Rap in JUNV infection suggests that autophagy promotes JUNV yield and increases viral progeny, which is consistent with the results of our autophagy inhibition assays.

### JUNV infection induces LC3 aggregation in fluorescent-tagged LC3 transfected cells

**Fig 5:**
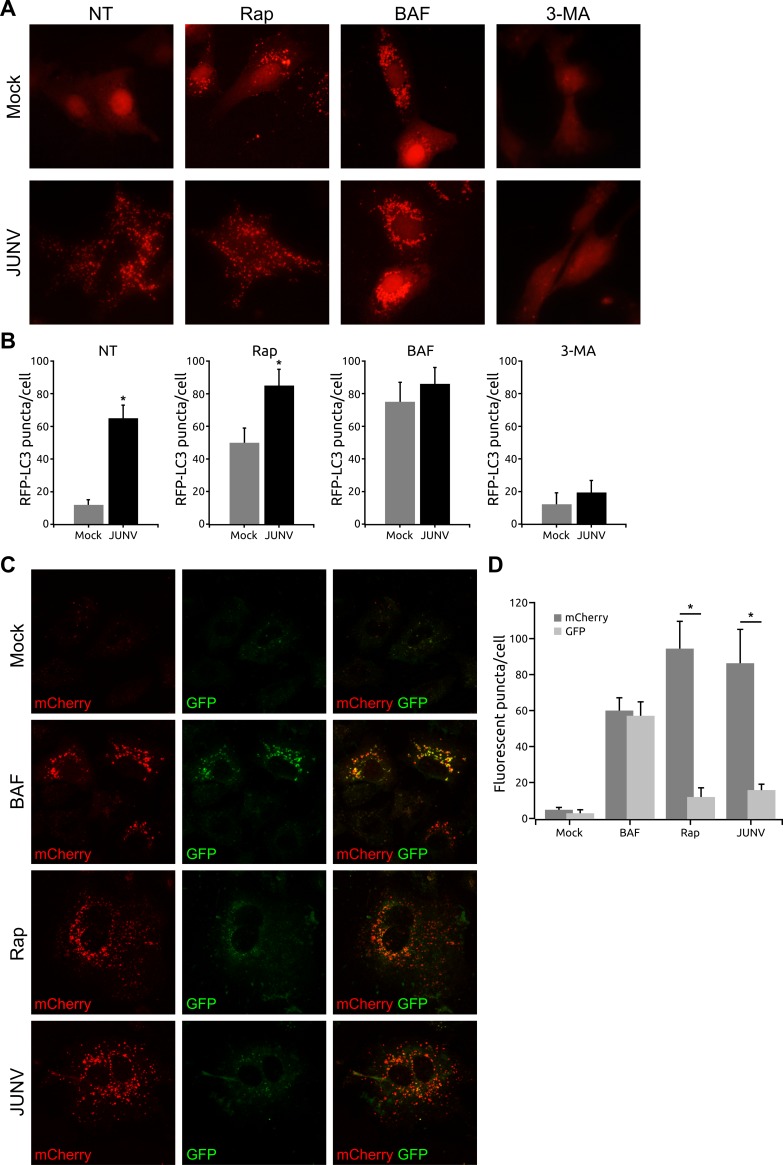
JUNV induces LC3 aggregation in RFP-LC3 and mCherry-GFP-LC3 transfected cells. A549 cells were transfected with RFP-LC3 plasmid for 24 h, followed by JUNV infection (control: mock infection). In parallel experiments, transfected cells were treated with rapamycin (Rap, 100 nM), bafilomycin A1 (BAF, 100 nM) or 3-methyladenine (3-MA, 5 mM) 3 h prior to infection. NT: no treatment. Cells were fixed (24 h p.i.), mounted and analysed by confocal imaging. (A) Autophagosome formation by LC3 aggregation (RFP-LC3 positive puncta) was observed by fluorescence microscopy and (B) the number of RFP puncta per cell was quantified using ImageJ software. Data were collected from >50 cells for each condition. (C) A549 cells were transfected with mCherry-GFP-LC3 plasmid and treated with rapamycin, BAF or infected with JUNV 24 h post-transfection. Cells were fixed and mounted for confocal microscopy (24 h p.i.). (D) Quantification of the number of red (mCherry) or green (GFP) fluorescent puncta in mCherry-GFP-LC3-transfected cells treated or JUNV infected as indicated in (C). Fifty cells were analysed per assay. The data correspond to the mean ± s.d. (n = 3); Student's t-test; *, P <0.05.

In order to provide further evidence of JUNV-induced autophagosome formation, cells were transfected with a plasmid expressing a red fluorescent protein-tagged LC3 (RFP-LC3), and redistribution of LC3 was established by fluorescence microscopy. After JUNV infection, a redistribution of RFP-LC3 from a diffuse (mock, no treatment) to a punctate pattern was apparent (Fig 5A and 5B), further confirming the increased formation of autophagosomes after viral infection. To evaluate the effect of cationic lipids (lipofection reagent) on autophagy induction and viral multiplication, we also performed RFP-LC3 transfection experiments using a cationic polymer (polyethylenimine, PEI) as transfection reagent. After 24 h, cationic lipids or polymer-transfected cells were infected with JUNV. No differences were detected in RFP-LC3 puncta/cell and virus yield regardless of the transfection reagent employed (S7 Fig). We also compared the effects of Rap, BAF and 3-MA on the generation of RFP-LC3 punctate pattern in infected A549 cells. The cells infected with JUNV presented similar numbers of puncta compared to mock-infected cells treated with Rap—commonly used as positive control—but the cells treated with Rap and JUNV-infected showed even higher values of puncta per cell than the corresponding control (Fig 5B). As expected, the number of puncta in mock-infected cells treated with BAF was higher than in the NT control, in agreement with previous reports indicating that the treatment with BAF promotes the accumulation of autophagosomes by blocking their fusion with lysosomes [43]. No significant difference was detected in the number of RFP-LC3 puncta per cell in mock versus infected cells pre-treated with BAF, although a change in the size and morphology of the induced puncta was observed. However, 3-MA reduces the formation of RFP-LC3 puncta in cells infected with JUNV when compared to non-treated infected cells (Fig 5B).

To analyse the status of the autophagy flux during JUNV infection, we used a tandem mCherry-GFP-tagged LC3 expression vector. As GFP is sensitive to the acidic conditions in the lysosome, only the fluorescence from mCherry persists in this environment. As a consequence, the colocalisation of mCherry and GFP signals indicates a vesicular compartment that has not fused with an acidic compartment as in the case of the phagophore or autophagosome. On the other hand, the red fluorescence of mCherry without GFP signal corresponds to an autophagosome fused with an endosome or lysosome, called amphisome or autolysosome, respectively [44]. A549 cells were transfected with mCherry-GFP-LC3 plasmid, and 24 h post-transfection were treated with rapamycin, BAF or infected with JUNV. When compared to control cells, an increase in the number of mCherry puncta following rapamycin treatment was observed, indicating promoted autophagy and fusion between autophagosomes and acidic compartments (Fig 5C). In contrast, the blockage of organelle acidification triggered by BAF resulted in the accumulation of LC3 puncta containing both, GFP and mCherry signal (Fig 5C and 5D). Cells infected with JUNV displayed an accumulation of mCherry puncta (Fig 5C and 5D), where only a few of them colocalised with GFP puncta. This result indicates that there is an accumulation of acidified autophagosomal structures in JUNV-infected cells and suggests that the virus induced both the initiation and maturation of autophagosomes but did not block the process at the autophagosome stage.

### The induction of autophagy is independent of viral replication

**Fig 6:**
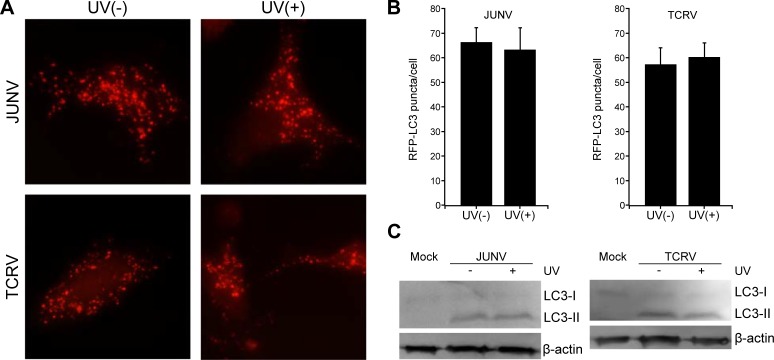
JUNV and TCRV induced autophagy is independent of viral replication. A549 cells were transfected with RFP-LC3 plasmid for 24 h, followed by infection with replication competent JUNV or TCRV or UV-inactivated JUNV or TCRV. (A) Autophagosome formation by LC3 aggregation (RFP-LC3 positive puncta) was observed by fluorescence microscopy and (B) the number of RFP puncta per cell was quantified using ImageJ software. Data were collected from >50 cells for each condition. (C) In parallel experiments, cells were lysed and analysed by WB with antibodies as indicated. Full-length blots are presented in S4 Fig. The data correspond to the mean ± s.d. (n = 3).

The activation of autophagy by JUNV infection could be caused either by incoming virions or by viral replication products. To determine whether JUNV replication is required for the induction of autophagy, we challenged RFP-LC3-transfected cells with either live JUNV or UV-inactivated JUNV and measured the effect on autophagy by monitoring aggregation of RFP-LC3 using fluorescence microscopy and the conversion of LC3-I to LC3-II by WB. Previously, we used the plaque formation assay to verify that the UV-inactivated virus was replication defective (S5 Fig). At 24 h post exposure to JUNV, there was no difference between the number of RFP-LC3 puncta per cell induced by UV-inactivated JUNV and replication competent JUNV (Fig 6A and 6B). Moreover, WB analysis revealed similar levels of LC3-II/LC3-I ratio in A549 cells treated either with UV-inactivated or replication competent JUNV (Fig 6C). Overall, these results suggest that JUNV particles play a role, but JUNV replication is not required for the induction of autophagosomes. We also evaluated the capacity of another NW arenavirus to activate autophagy by performing similar experiments with Tacaribe virus (TCRV) as shown in Fig 6. The RFP-LC3 punctate pattern and the LC3-II level induced by TCRV infection closely resembled the results observed with JUNV infected cells (Fig 6A–6C).

### JUNV induces colocalisation of LC3 and p62, ATG16, RAB5, RAB7A or LAMP1

**Fig 7:**
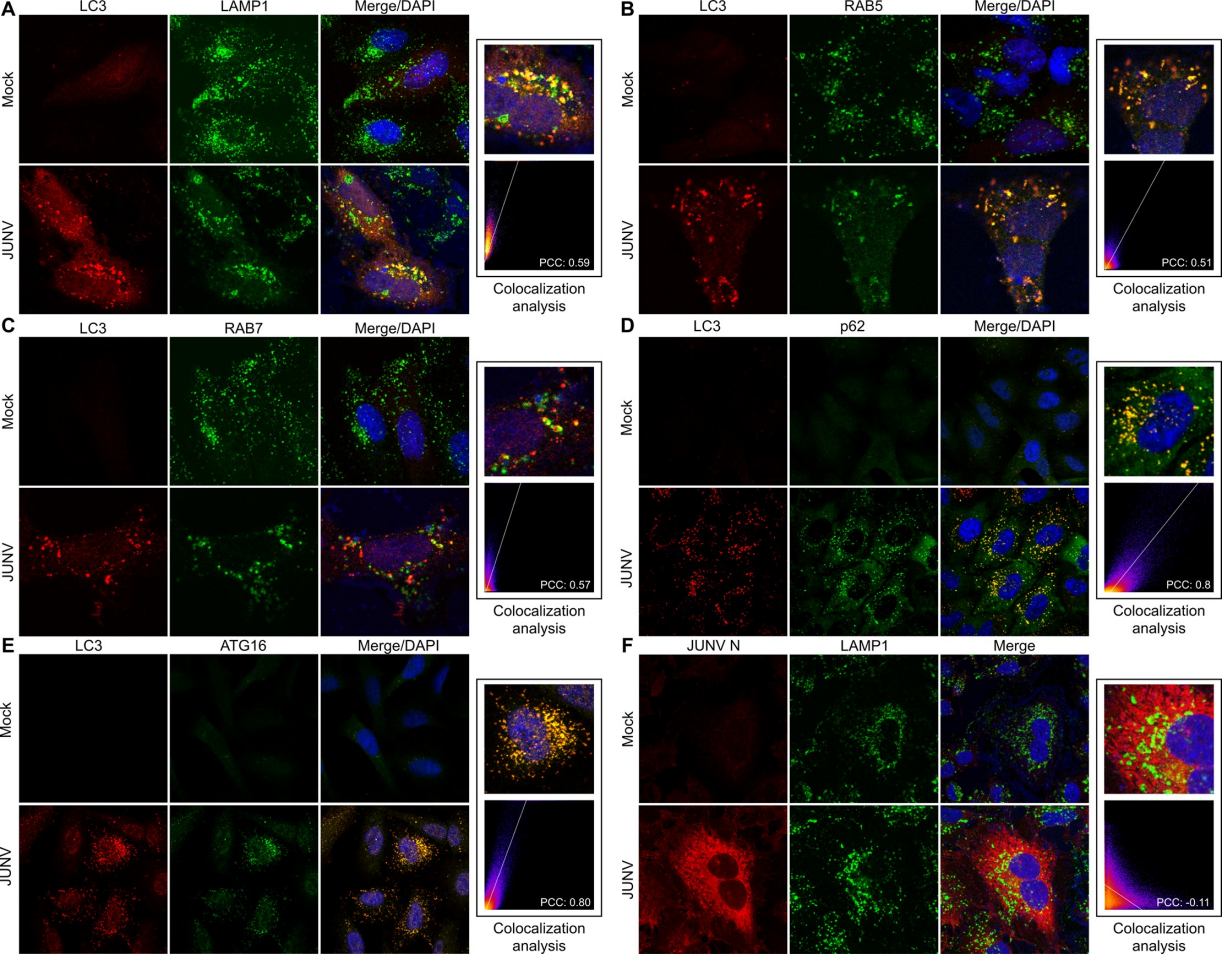
JUNV induces the colocalisation of p62, ATG16, RAB5, RAB7A and LAMP1 with the autophagosomal LC3 protein. A549 cells were mock or JUNV infected. After 24 h p.i, the cells were fixed, permeabilised and incubated with the primary Abs anti-LC3 and anti-LAMP1 (A), anti-RAB5 (B), anti-RAB7A (C) anti-p62 (D) or anti-ATG16 (E). Antibodies conjugated to Alexa488 (LAMP1, RAB5 and RAB7A) and Cy3 (LC3) were used as secondary Abs. Colocalisation of LAMP1 and JUNV N in infected cells was assessed by immunoconfocal microscopy analysis using Abs anti-LAMP1 and anti-JUNV N as primary Abs, and Abs conjugated to Alexa488 (LAMP1) and Cy3 (N) as secondary Abs (F). The nuclei were stained with DAPI. Representative images of confocal microscopy and magnification of a small area are shown. Images were analysed by using Image J software. Pearson’s coefficients were used for assessing colocalisation.

Upon maturation, autophagosomes may fuse with late endosomes and lysosomes, forming autolysosomes. We examined virus-infected cells for the association between autophagosomes (LC3) and early endosomes (RAB5), late endosomes (RAB7A) or the lysosomal-associated membrane protein 1 (LAMP1). As demonstrated in Fig 7A and 7B, the colocalisation of LC3 and early and late endosomal markers was detected in JUNV-infected cells, while uninfected cells exhibited almost no overlap. Moreover, JUNV-induced vesicles are positive for both LC3 and LAMP1, indicating that these vesicles have likely fused with lysosomes (Fig 7C). No colocalisation of viral N and LAMP1 was observed in infected cells (Fig 7F). LC3 also colocalised with ATG16L1 and p62 in JUNV infected cells, which represent isolation membrane (Fig 7D and 7E). These results suggest that JUNV infection induced the formation of novel autophagosomes without alteration of their maturation.

### Depletion of endogenous ATG5, ATG7, Beclin 1 and RAB7A reduces viral yield

**Fig 8:**
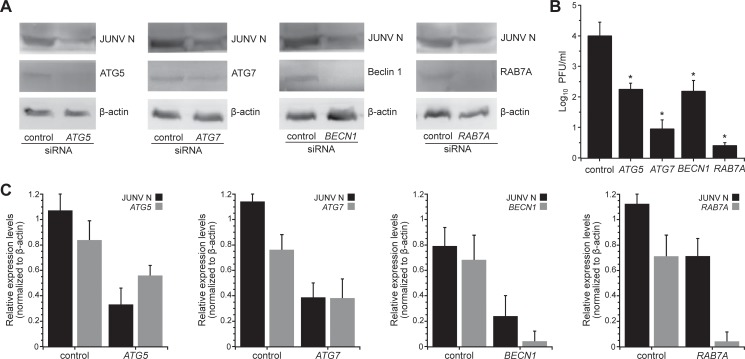
Depletion of ATG5, ATG7, Beclin 1 or RAB7A by siRNA reduces JUNV yield. A549 cells were transfected with siRNA oligonucleotides targeting the indicated gene. After 24 h, the cells were infected with JUNV. (A) Western blot analysis of JUNV N, ATG5, ATG7, Beclin 1, RAB7A and β-actin expression in JUNV infected cells transfected with the indicated siRNAs. Full-length blots are presented in S6 Fig. (B) Plaque assay results for scrambled (control) or *ATG5*, *ATG7*, *Beclin 1* and *RAB7A* siRNA transfected cells 24 h p.i. (means ± s.d.; n = 3; Student's t-test; *, P <0.05). (C) JUNV N/β-actin, ATG5/β-actin, ATG7/β-actin, Beclin1/ β-actin and RAB7A/β-actin expression ratio was established by densitometry using ImageJ software. The data correspond to the mean ± s.d. (n = 3); Student's t-test; *, P <0.05.

In addition to the studies with pharmacological agents, we used siRNA targeting key autophagy genes, *Beclin 1*, *ATG5*, *ATG7* and *RAB7A*, in order to evaluate the role of these cellular factors on viral yield. As mentioned earlier, Beclin 1 is part of class III PI3K complex involved in the initiation of autophagy [45]. ATG5 contributes to LC3-PE conjugation, while ATG7 acts as an E1-like enzyme for the ubiquitin-like proteins ATG12 and LC3, making both essential regulators of autophagosome assembly [46]. Finally, RAB7A has a crucial role in the final maturation of autophagosomes into autolysosomes [47,48]. To deplete these proteins, we treated A549 cells with siRNA for 24 h and then challenged the cells with JUNV. WB analysis of cell lysates revealed that levels of endogenous ATG5, ATG7, Beclin 1 or RAB7A protein were significantly reduced in cells treated with the specific siRNAs compared to scrambled siRNA (Fig 8A and 8C). In parallel, we observed a decrease in the expression of N in JUNV infected cells treated with autophagy-specific siRNA compared to the control siRNA (Fig 8A and 8C). Also, depletion of autophagic proteins led to reduced viral titres (Fig 8B). Viral load in the supernatant medium of infected cells declined by 49%, 44%, 76% and 90% after silencing Beclin 1, ATG5, ATG7 and RAB7A, respectively. Collectively, the data demonstrate that inhibition of autophagy affects JUNV yield.

## Discussion

In the present work, we studied the interaction between the cellular autophagy pathway and the NW arenavirus JUNV. LC3 puncta were detected by immunostaining for endogenous LC3 or following the redistribution of mCherry-GFP-LC3 or RFP-LC3 signal after infection. This, coupled with the observation that LC3-I was converted to LC3-II, suggests that the LC3 puncta induced by JUNV were autophagosomes. In addition, our results show that JUNV infection promoted p62 degradation, indicating that complete autophagic flux was triggered upon JUNV infection. Moreover, increasing the number of autophagosomes prior to viral infection by rapamycin treatment promoted viral yield. Finally, we observed colocalisation of JUNV N, the major nucleocapsid structural protein, and LC3, indicating that viral proteins were associated with autophagic structures.

We further showed that the pharmacological modulation of autophagy altered the outcome of JUNV multiplication. Class III PI3K plays a pleiotropic role in autophagy and protein sorting pathways [49]. The compound 3-MA blocks the formation of autophagosomes by inhibiting the activity of class III PI3K. Our data demonstrate that 3-MA affects JUNV yield, suggesting that an early step in the formation of the autophagosome such as recruitment of single-membrane secretory pathway-derived vesicles or newly formed double-membrane vesicles could provide a site for RNA replication. Moreover, 3-MA reduces virus capacity to induce RFP-LC3 puncta suggesting that autophagosomes induced by JUNV require class III PI3K activity, and consequently, their formation may be dependent on the Beclin 1-VPS34 complex. In agreement with our results, Linero and Scolaro found that JUNV infection activates PI3K/Akt signalling pathway at an early stage of infection [24]. In fact, UV-inactivated JUNV infected cells redeemed the pattern of Akt phosphorylation observed for the replication-competent virus, indicating that PI3K/Akt signalling is triggered at an early stage of the viral cycle [24]. Similarly, we observed that UV-inactivated virus induces a RFP-LC3 punctate pattern comparable to infectious virus, suggesting that the autophagosome formation induced by JUNV would be triggered by early events of the virus-cell interaction. Other viruses also activate autophagy independently of viral replication as is the case of foot-and-mouth disease virus [50], vesicular stomatitis virus [51] and human cytomegalovirus [52]. In addition, the non-canonical Raf/MEK/ERK signalling pathway, which modulates autophagy by regulating Beclin 1 [53–55], could participate in early events during JUNV-induced autophagy. Supporting this idea, the activation of the Raf/MEK/ERK signalling pathway induced by JUNV infection is also required to ensure efficient JUNV replication [56,57]. Even though inhibition of ERK1/2 signalling pathway does not affect JUNV adsorption, internalisation and uncoating [57], we cannot exclude that early events that take place after uncoating but before viral replication, such as sensing of viral cargo molecules, may activate this pathway, and in consequence regulate autophagy.

We demonstrated that silencing critical genes of the different stages of autophagy, namely *ATG5*, *ATG7*, *Beclin 1* or *RAB7A*, reduces viral protein expression and viral progeny. Impaired virus multiplication after ATG7 knockdown, an essential regulator of autophagosome assembly, has been observed for various RNA viruses, including coxsackievirus B3, HCV and rotavirus [58–60]. In the following steps of autophagosome biogenesis, ATG5, ATG12, and ATG16 form the autophagic elongation complex determining the site of LC3 lipidation [61]. In particular, the contribution of ATG5 to viral replication has been demonstrated for several RNA viruses [62–64]. More recently, Fahmy and Labonté [65] found that the autophagy elongation complex is recruited at the membranous web—an intracellular membrane rearrangement composed of double-membrane vesicles—and promotes HCV replication and assembly. Likewise, we found that JUNV induces the colocalisation of LC3 with p62 and ATG16, suggesting the formation of the autophagy elongation complex. Moreover, ATG5 interacts transiently with the HCV RNA polymerase as a proviral factor during the onset of viral infection [64]. Another autophagy component evaluated in this study—Beclin 1—sits at the core of autophagy regulation [66,67]. The knockdown of Beclin 1 significantly reduced the yield of JUNV, similarly to other viruses such as HCV [59] and influenza A virus [68]. Finally, the small GTPase RAB7A is considered a multifunctional regulator of autophagy and endocytosis [48,69]. RAB7A controls the maturation of endosomes and autophagosomes, regulates the trafficking of endosomal multivesicular bodies to lysosomes and participates in the fusion step with lysosomes. It has been shown that individual knockdown of LAMP2 and RAB7A, which impairs complete autolysosome maturation, inhibits both HCV RNA replication and viral protein expression [70]. Our results showing that JUNV infection induces the colocalisation of RAB5, RAB7A or LAMP1 and LC3, indicate that phagophore or autophagosome fusion with early and late endosomes is not being blocked by JUNV infection. On the other hand, silencing of RAB7A had a strong inhibitory effect on viral multiplication, reflecting the role of endosomal trafficking on viral infection outcome. Other studies performed with the NW arenavirus Pichindé and JUNV, have shown that viral entry is trafficked through the clathrin- and dynamin-2-mediated endocytosis, by which the virus travels through RAB5-mediated early endosomes and RAB7A-mediated late endosome [22,71]. Although the silencing of RAB7A is expected to affect the uncoating of JUNV, we can not rule out that late fusion events essential for the production of viral infectious particles are also compromised. Further experiments are needed to dissect the effect of RAB7A on JUNV multiplication.

Based on several studies describing the life cycle of RNA viruses, Nunberg *et al*. [72] proposed that arenavirus replication was likely to be compartmentalised to specialized virus-induced organelles referred to as replication-transcription complexes (RTCs). They found that Candid#1, the vaccine attenuated strain of JUNV, and TCRV RNA synthesis takes place in discrete cytosolic puncta, the formation of which is induced in part by N and suggests that RTCs also include a membrane component. Although we did not detect RTCs or RNA directly, the fact that we observed colocalisation of N and LC3 in a similar cytoplasmic punctate pattern suggests that autophagosome membranes could be involved in RTC assembly. However, these authors did not find colocalisation of RTCs induced by TCRV and autophagosomes [72]. This alleged discrepancy could probably be related to immunostaining methods or even the use of different cell line and virus. Further studies will be needed in order to establish the relationship between the LC3-N puncta and RTCs induced by JUNV infection. It is also likely that the autophagosome provides a physical scaffold where JUNV may be assembled or viral RNA synthesised. Recruitment of components of the autophagic machinery to assemble viral RTC could be involved in both mechanisms. For example, rotavirus initiates autophagy and hijacks this membrane trafficking pathway to transport viral proteins to viroplasms [73]. A recent study shows that LCMV RNA aggregates colocalise with the early endosomal marker RAB5c and the viral glycoprotein in infected cells, and propose that LCMV uses the surface of a cellular membrane-bound organelle as a site for the pre-assembly of viral components [74].

For some viruses, the events related to vesicular acidification are used to assist with their multiplication. In particular, JUNV requires a low pH in the entry vesicle for the success of the glycoproteins-mediated membrane fusion [23,75]. That is consistent with entry by clathrin-mediated endocytosis, as clathrin-coated vesicles deliver their cargo into endosomes with an acidic content [21]. In agreement with previous studies [76], the pre-treatment with lysosomotropic agents such as BAF and NL severely affects viral yield in A549 cells. Nevertheless, when these agents were added after the early steps of the viral cycle (binding, receptor-mediated endocytosis, uncoating and genome release), the viral yield was still affected. These results suggest that vesicle acidification also plays a role in the late events of the viral cycle. In the experiment performed by Castilla *et al*. [76], Vero and BHK cells were used as a model to assess the effect of BAF and concanamycin A (CA), another specific inhibitor of vacuolar type-ATPase, in the production of JUNV infectious particles. Even when CA was added 5 h p.i., both, extracellular virus titre and infectious cell-associated virus quantified 24 h p.i., were significantly reduced. Castilla *et al*., suggest that CA could also interfere with the egress of cell-associated infectious virus to the extracellular medium besides its early effect on viral endocytosis. These results are in agreement with our experimental findings using BAF (pre vs post-infection experiments, Fig 4). For some viruses like poliovirus, vesicle acidification promotes maturation of the assembled, encapsidated virus particles into infectious virions [77]. In the case of JUNV, we can speculate that late membrane fusion events related to the viral assembly and budding, as could be the fusion of autophagosomes and MVBs or the plasma membrane, are also affected by post-infection treatment with BAF.

In summary, our findings suggest that JUNV activates autophagy in A549 human cells favouring viral multiplication. To our knowledge, this is the first report showing that the infection of an arenavirus induces autophagy. Our studies provide novel insights into arenavirus-host interactions and open a new window to examine the pathogenesis of this viral family. This knowledge could contribute to the development of antiviral strategies against arenavirus infection.

## Supporting information

S1 Fig Full-length blots of Fig 1. Red dashed lines show the cropping area. The brightness of the whole image was adjusted during processing of the blots.
(EPS)

S2 Fig Full-length blots of Fig 3. Red dashed lines show the cropping area. The brightness of the whole image was adjusted during processing of the blots.
(EPS)

S3 Fig Full-length blots of Fig 4. Red dashed lines show the cropping area. The brightness of the whole image was adjusted during processing of the blots.
(TIF)

S4 Fig Full-length blots of Fig 6. Red dashed lines show the cropping area. The brightness of the whole image was adjusted during processing of the blots.
(TIF)

S5 Fig UV inactivation of JUNV and TCRV. Residual infectivity of UV-inactivated JUNV or TCRV was assessed by plaque formation assay. Samples from the viral stocks and the UV-irradiated virus were 10-fold diluted and titrated in Vero E6 cells. Representative microscopy images of the plates (24-well) are shown. No viral plaques were observed in the UV-irradiated stocks. NT: no treatment.
(TIF)

S6 Fig Full-length blots of Fig 8. Red dashed lines show the cropping area. The brightness of the whole image was adjusted during processing of the blots.
(TIF)

S7 Fig JUNV induces LC3 aggregation in RFP-LC3 in A549 transfected cells using both cationic polymers or lipids. A549 cells were transfected with RFP-LC3 plasmid using polyethylenimine (PEI) or Lipofection reagent (LR, Roche). After 24h, transfected cells were mock or JUNV infected, and analysed 24 h p.i. (A) Autophagosome formation by LC3 aggregation (RFP-LC3 positive puncta) was observed by fluorescence microscopy and (B) the number of RFP puncta per cell was quantified using ImageJ software. (C) Determination of viral titre of the cell supernatant from experiments performed as indicated before by plaque formation assays (PFU/ml) on Vero E6 cells. NT: not transfected A549 infected cells. The data correspond to the mean ± s.d. (n = 3); Student's t-test; *, P <0.05.
(EPS)
