# Supplementary figures and images for: Junín virus induces autophagy in human A549 cells
Source: PLoS One. 2019 Jun 19;14(6):e0218730. doi: 10.1371/journal.pone.0218730 (PMC6583977; doi:10.1371/journal.pone.0218730)

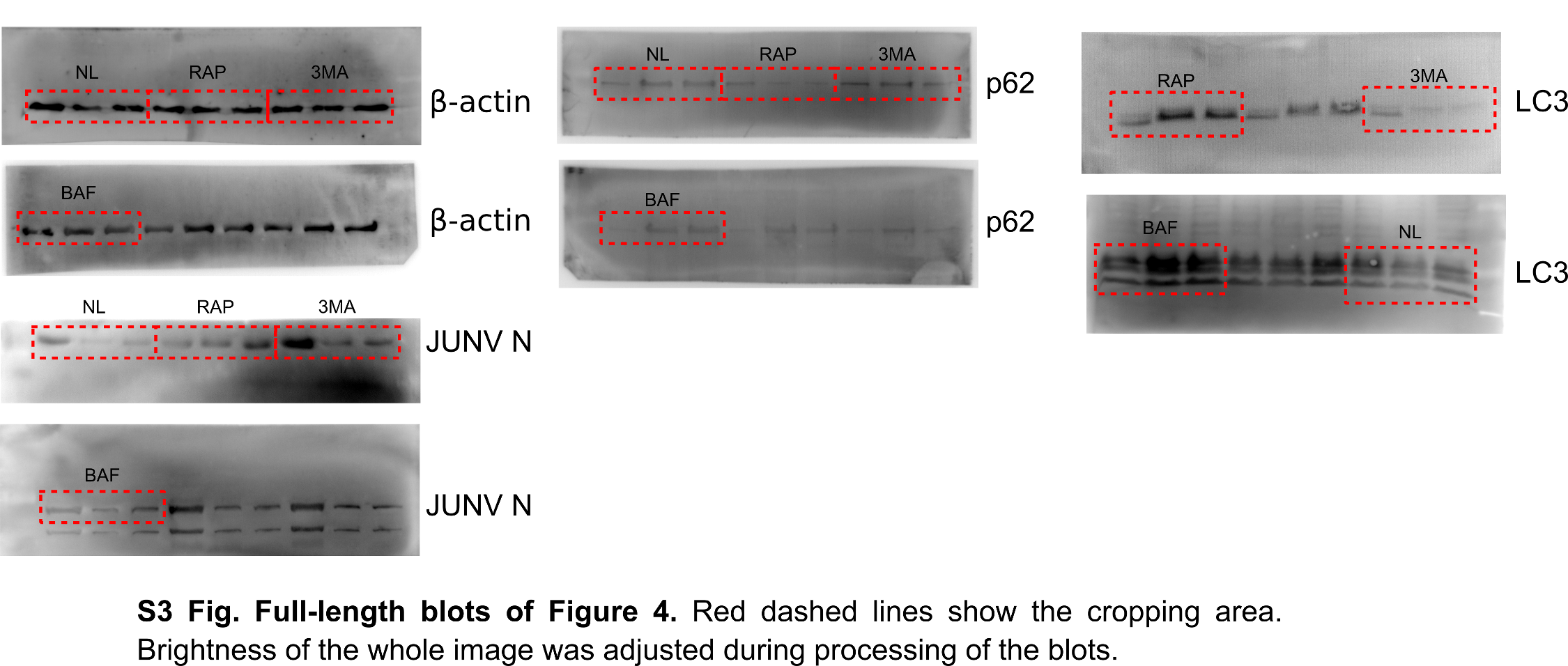

Supplement: S3 Fig — Red dashed lines show the cropping area. The brightness of the whole image was adjusted during processing of the blots. (TIF) [file pone.0218730.s003.tif]

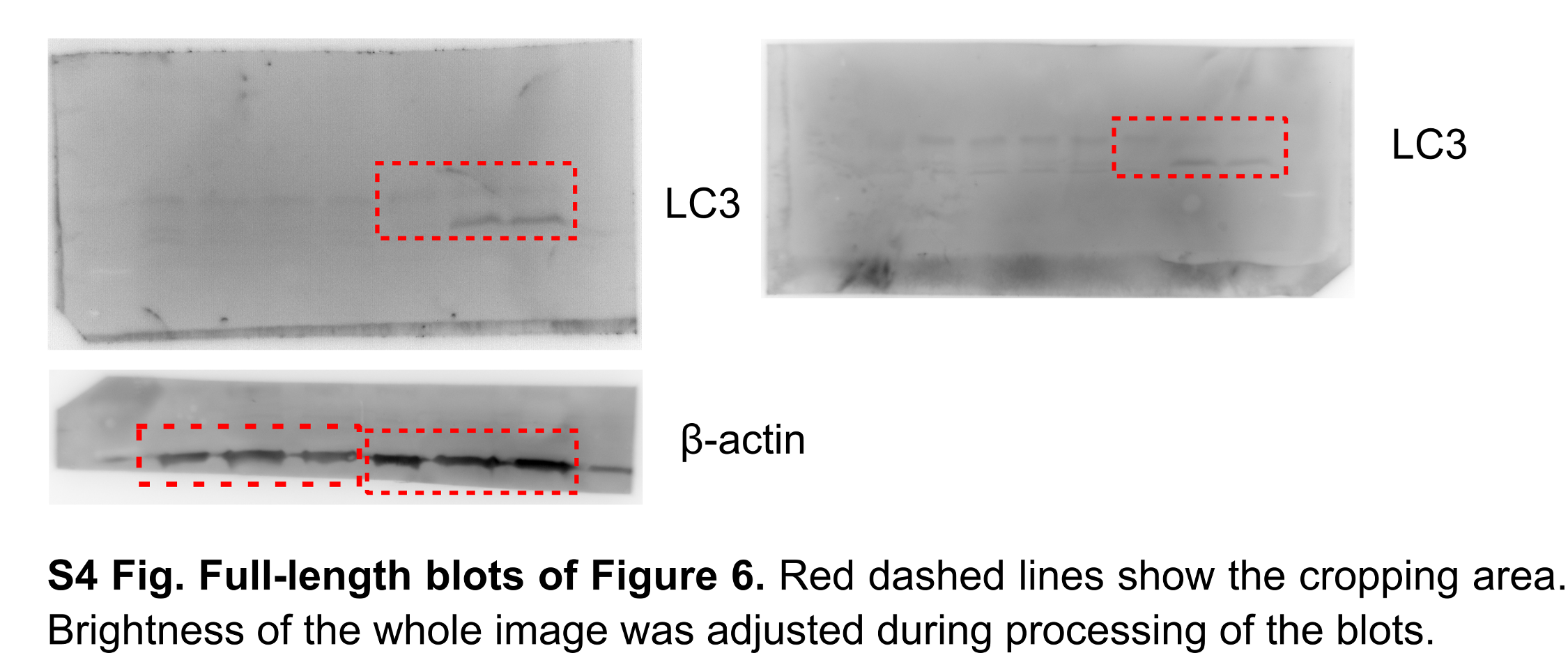

Supplement: S4 Fig — Red dashed lines show the cropping area. The brightness of the whole image was adjusted during processing of the blots. (TIF) [file pone.0218730.s004.tif]

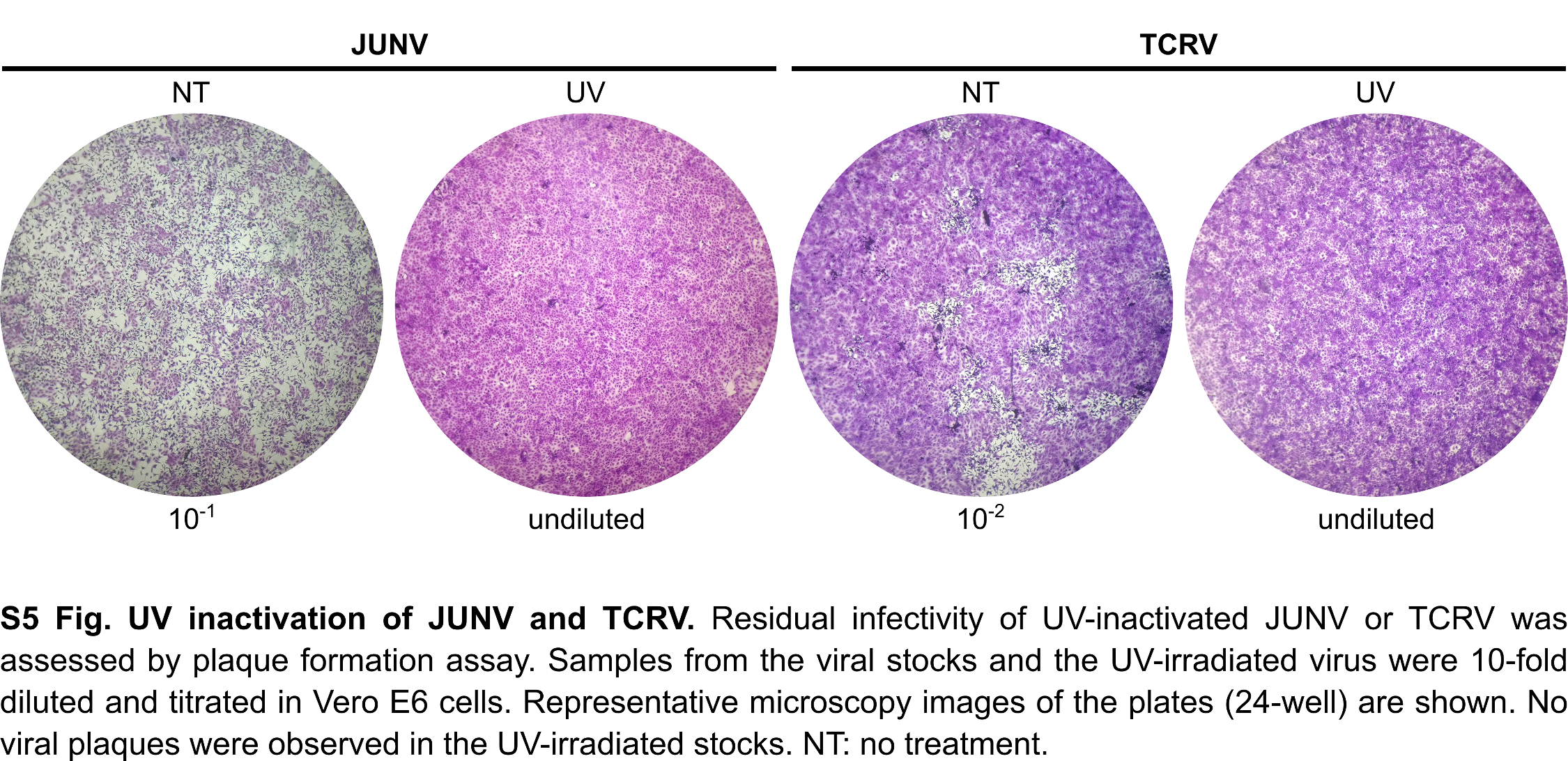

Supplement: S5 Fig — Residual infectivity of UV-inactivated JUNV or TCRV was assessed by plaque formation assay. Samples from the viral stocks and the UV-irradiated virus were 10-fold diluted and titrated in Vero E6 cells. Representative microscopy images of the plates (24-well) are shown. No viral plaques were observed in the UV-irradiated stocks. NT: no treatment. (TIF) [file pone.0218730.s005.tif]

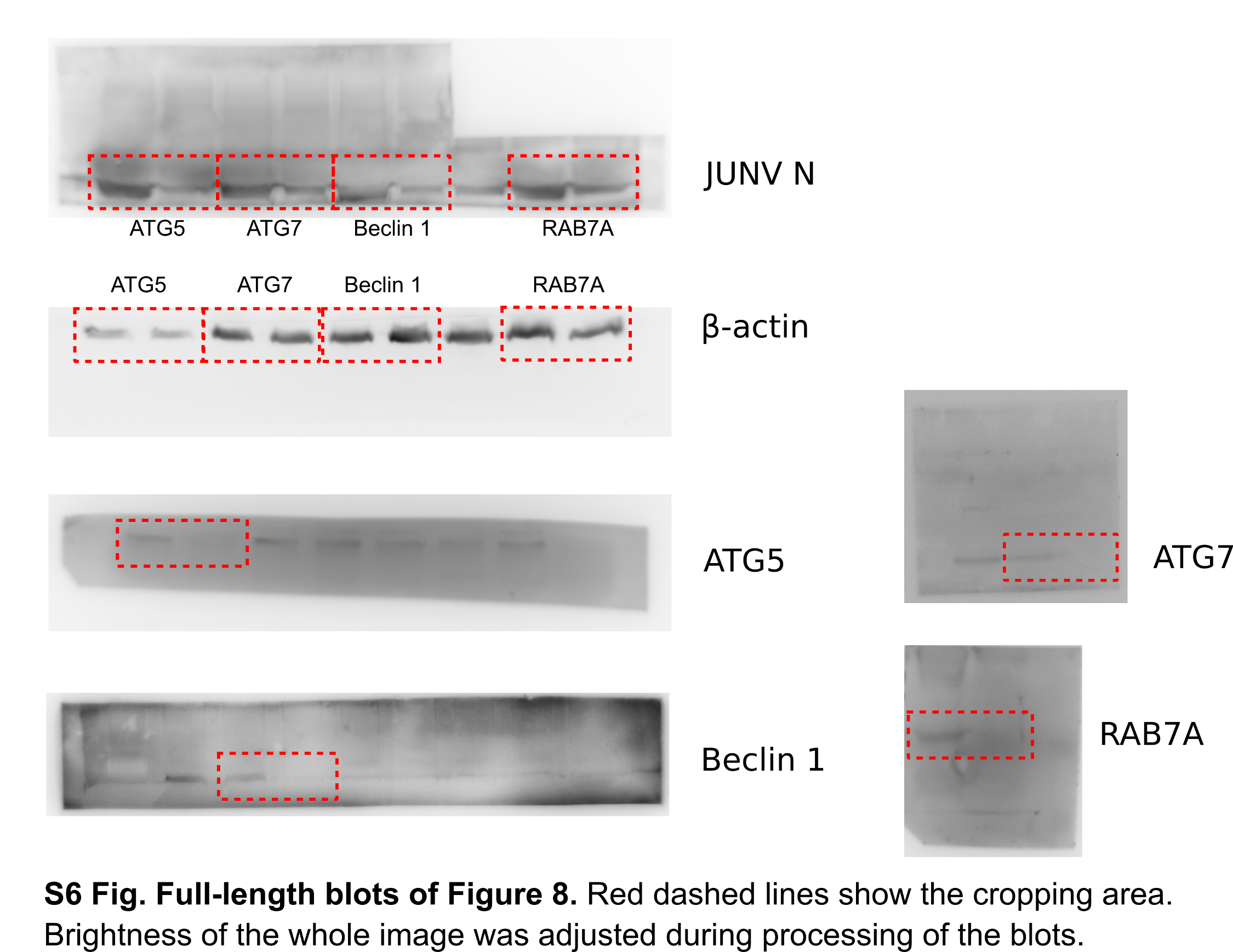

Supplement: S6 Fig — Red dashed lines show the cropping area. The brightness of the whole image was adjusted during processing of the blots. (TIF) [file pone.0218730.s006.tif]
